# Localized Surface Plasmon Resonance as a Biosensing Platform for Developing Countries

**DOI:** 10.3390/bios4020172

**Published:** 2014-06-20

**Authors:** Jules L. Hammond, Nikhil Bhalla, Sarah D. Rafiee, Pedro Estrela

**Affiliations:** Department of Electronic and Electrical Engineering, University of Bath, Bath BA2 7AY, UK; E-Mails: J.L.Hammond@bath.ac.uk (J.L.H.); N.Bhalla@bath.ac.uk (N.B.); S.Rafiee@bath.ac.uk (S.D.R.)

**Keywords:** localized surface plasmon resonance, low-cost biosensors, medical diagnostics, environmental monitoring

## Abstract

The discovery of the phenomena known as localized surface plasmon resonance (LSPR) has provided the basis for many research areas, ranging from materials science to biosensing. LSPR has since been viewed as a transduction platform that could yield affordable, portable devices for a multitude of applications. This review aims to outline the potential applications within developing countries and the challenges that are likely to be faced before the technology can be effectively employed.

## 1. Introduction

A staggering 80% of the population currently live in developing countries. Such countries are considered to have low living standards, weak industry and a low human development index (HDI)—a measure of poverty, literacy, education and life expectancy.

In 2000, a series of UN Millennium Development Goals were outlined by 189 countries in an attempt to improve the global quality of life [1]:

- combat diseases, such as HIV/AIDS and malaria; halt and begin to reverse cases by 2015;
- reduce maternal mortality ratio by three-quarters by 2015;
- reduce child mortality by two-thirds by 2015;
- reduce the proportion of people who suffer from hunger by halve by 2015;
- integrate the principles of sustainable development and reverse the loss of environmental resources;
- halve the proportion of people without sustainable access to safe water and sanitation by 2015.

Whilst considerable progress has been achieved in meeting the goals to reduce poverty and provide drinking water, as indicated in the UN 2013 report [2], accelerated progress and bolder action are needed in many areas. Increased efforts are still required in order to come close to meeting the 2015 targets.

The quality of life can be considered to be dependent on the control of diseases, agricultural quality and the quality of our environment. Hence, it has become an increasingly important motivation to develop low-cost, portable devices that can provide fast, reproducible, sensitive and selective sensing assays for such applications. For developing countries with inadequate health services, inefficient agricultural productivity and apathy towards environmental sustainability, the need for such devices is even more prominent. 

Many current sensing techniques are inhibited by complicated labelling procedures, the requirement for large sample volumes and expensive, complex instrumentation. Localized surface plasmon resonance (LSPR) sensors offer high sensitivity [3], a small footprint [4] and are reasonably affordable due to the simplicity of instrumentation [5]. 

LSPR has already been shown to be an effective platform for the detection of biomolecules [6] and the sensing technique offers the advantage of being easily multiplexed to enable high throughput screening in an array format [7]. Most importantly, the detection of concentrations down to the zeptomole range [8] has been demonstrated, substantiating the high sensitivity that can be achieved. 

It is these inherent characteristics that make LSPR such an interesting proposition as a platform to develop effective and economical sensors that could be used to improve the quality of life in developing countries. Whilst several good LSPR reviews exist [9,10,11,12,13,14,15], in this review, we focus on relevant work that underpins the potential applications in developing countries and discuss some of the factors that need to be addressed in order for such a platform to be effectively commercialised. 

### 1.1. LSPR Principle

LSPR is an optical phenomenon that causes a collective oscillation of valence electrons and subsequent absorption within the ultraviolet-visible (UV-Vis) band, due to interactions between the incident photons and the conduction band of a noble metal nanostructure. The wavelength-selective absorption can display molar extinction coefficients as large as 3 × 10^11^ M^−1^·cm^−1^ [16].

**Figure 1:**
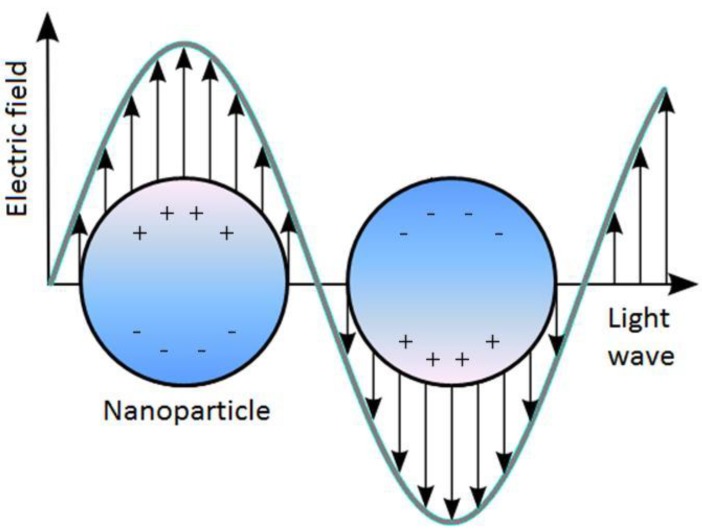
Schematic of localized surface plasmon resonance (LSPR) where the free conduction electrons in the metal nanoparticle are driven into oscillation due to strong coupling with incident light.

LSPR differs from SPR as the induced plasmons oscillate locally to the nanostructure (see Figure 1), rather than along the metal-dielectric interface. As a result, the decay length of the electromagnetic field observed in surface plasmon is in the order of 200 nm [17], whereas the decay length of the electromagnetic field in localized surface plasmons is in the order of 6 nm [18] (both decaying exponentially). The shorter field decay length for LSPR reduces the sensitivity to interference from solution refractive index fluctuations whilst providing increased sensitivity to refractive index changes on the surface. This property acts as the foundation for biosensing applications.

LSPR can be elegantly described by Mie’s solution to Maxwell’s equation, occurring as a consequence of the restriction to the movement of electrons through the internal lattice of a noble metal when the size of the noble metal structure is scaled down to the nano level (<100 nm) [19].

Equation (1) [20] demonstrates the dependence of extinction, *E*(*λ*), on the dimension, shape, density and local environment of the nanostructure:

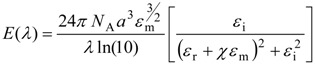
(1)
where *N*_A_ is the areal density of the nanostructure, *a* is the radius of the nanostructure (the nanostructure is modelled as a sphere), *ε*_m_ is the dielectric constant of the medium surrounding the nanostructure (the dielectric constant is assumed to be a positive, real integer and wavelength independent), *λ* is the wavelength of the absorbing radiation, *ε*_i_ is the imaginary portion of the nanostructure’s dielectric function, *ε*_r_ is the real portion of the nanostructure’s dielectric function and χ is the term that is used to describe the aspect ratio of the nanostructure [5].

LSPR spectroscopy offers sensing through transduction of refractive index changes in close proximity to the surface of the noble metal nanostructure. Formation of adlayers and biorecognition events on the surface cause quantifiable shifts in the LSPR extinction wavelength maximum, *λ*_max_, due to the dependence of the adlayer refractive index, *n* (the refractive index is related to the dielectric constant by *n* = *ε*^1/2^), as demonstrated by Equation (2):

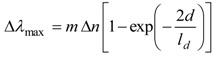
(2)
where *m* is the bulk refractive index response of the nanostructure, *d* is the effective adsorbate layer thickness and *l_d_* is the characteristic electromagnetic field decay length, modelled as an exponential decay [21]. The binding kinetics can then be monitored by tracking ∆*λ*_max_ as a function of either time or analyte concentration [22].

#### 1.1.1. Sensing Modes

There are two main sensing modes that can be implemented. In the first mode, UV-Vis spectroscopy is used to monitor wavelengths of light that cause the collective oscillation to occur; this mode is often referred to as ‘wavelength-shift sensing’. Here, changes in wavelength extinction curves can be monitored as a function of changes in the adlayer refractive index caused by target analyte adsorption. 

The second mode utilises the formation of enhanced electromagnetic fields that extend from the nanostructure surface. This is the basis for all surface-enhanced spectroscopies, such as surface-enhanced Raman scattering (SERS) and surface-enhanced Raman resonance scattering (SERRS). The SERS enhancement factor is a result of enhancing both the incident excitation and resulting Stoke’s shifted Raman electromagnetic fields [23]. The inelastic (Raman) scattering of photons by molecules attached on a plasmonic substrate is significantly enhanced, and the scattered photons either lose or gain energy equivalent to the molecular resonance of the probed substrate. The surface-enhanced techniques offer the detection of a target analyte by monitoring changes in the enhanced electromagnetic fields.

As an adlayer develops on the substrate, it interacts with the surrounding local electromagnetic fields and enhances the Raman scattering by a factor of 10^6^ to 10^8^ for periodically distributed molecules and by as much as 10^14^ to 10^16^ for single molecules [9]. Maximum enhancement is achieved when the LSPR wavelength falls between the excitation wavelength and the wavelength of the scattered photon. This dependence of SERS on LSPR wavelength is complementary for molecular binding and identification studies.

#### 1.1.2. LSPR Dependence on Nanostructures

Several elements have been shown to support localized surface plasmons, including palladium (Pd), platinum (Pt), gold (Au) and silver (Ag). Both Au and Ag nanostructures are ubiquitous in the literature, with their popularity stemming from their high values of refractive index sensitivity (*m*) of 44 nm/refractive index units (RIU) [24] and 161 nm/RIU [25], respectively. Although Au nanostructures have a lower sensitivity than Ag nanostructures, they are more frequently selected for biosensing applications, due to their lower toxicity [26] and being less prone to oxidation [27].

**Figure 2:**
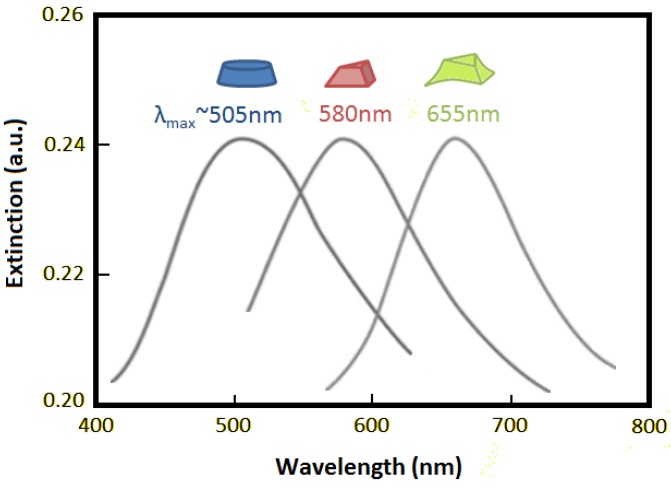
Diagram illustrating how the nanostructure shape affects the extinction wavelength maximum (λ_max_). Adapted from [9].

LSPR has been experimentally shown to be highly sensitive to several structural factors, such as dimension, shape and spacing [28], and this imposes a requirement in the fabrication of the sensor substrates for reliable and repeatable measurements. Figure 2 illustrates the effect that the nanostructure shape can have on the extinction wavelength maximum (λ_max_). 

Another important aspect to consider is the diameter of the nanostructure; for example, when the diameter of Au nanostructures is increased from ∼10 nm to ∼100 nm, λ_max_ has been shown to shift from ∼520 nm to ∼580 nm [29]. Hence, the diameter of the nanostructure will depict the wavelength at which the LSPR signal will be observed. 

Nanostructures of larger diameters also have the effect of producing broader spectra, yielding lower sensitivity [30]. A broader spectrum is caused by the dominance of non-radiative decay causing absorption (as opposed to scattering when radiative decay becomes dominant) [31]. This peak broadening can be classified as either homogeneous or inhomogeneous. Intrinsic properties of the nanostructure cause homogeneous broadening [32], whereas inhomogeneous broadening is the result of an averaging of the individual spectra from a collection of differing nanostructures [33].

#### 1.1.3. Optical Geometry

**Figure 3:**
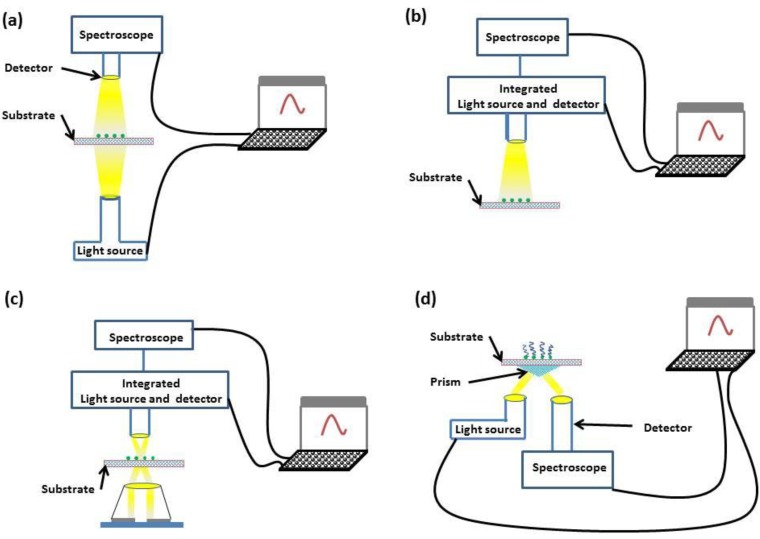
Diagrams of transmission (**a**), reflection (**b**), dark-field scattering (**c**) and TIR (**d**) geometries.

The optical geometries that are employed within LSPR sensors are transmission [34], reflection [35], dark-field scattering [36] and total internal reflection (TIR) [37]. These optical geometries are illustrated in Figure 3.

Both transmission and reflection geometries offer simplicity, with incident light interrogating the sample and either transmitted or reflected light being monitored through spectroscopy. If a greater dynamic range is required, dark-field scattering or TIR geometries can be used. In the dark-field scattering geometry, a high-numerical aperture condenser is used to illuminate the sensor substrate, and a low-numerical aperture objective collects the scattered light. In comparison, the TIR geometry places no numerical restriction on the objective aperture through the use of a prism coupler.

## 2. Applications

Much of the work to date has primarily focussed on the fabrication of novel nanostructures and the assessment of their properties; as a consequence, biosensing with LSPR platforms has very much remained in a state of proof of concept. Nevertheless, here, we cover a range of literature that demonstrates the potential of LSPR technology in medical, agricultural and environmental monitoring applications that can be considered relevant to tackling the issues in developing countries.

### 2.1. Medical

One of the main applications of LSPR in biosensing is the detection of small molecules. As bioreceptors (e.g., enzymes, antigens and antibodies) have dimensions in the range of 2–20 nm, similar to those of nanostructures, the two can be considered structurally compatible. This dimensional compatibility means that highly miniaturised signal transducers can be achieved through the combination of nanostructure characteristics, a wide selection of available bioreceptors and the rapid development of surface biofunctionalisation strategies [38]. The first step in the design of a LSPR biosensor is forming nanostructure-biomolecule conjugates. Noble metal nanostructures have unique photonic, electronic and catalytic properties. By functionalising these nanostructures with biomolecules, such as proteins or DNA, novel substrates can be developed to be used in different biomedical applications, such as sensing, imaging, diagnosis and therapy [39]. 

A plethora of biomolecules have been immobilised on nanostructures, such as: proteins, enzymes, peptides, antibodies, antigens, biotin, streptavidin, oligonucleotides and aptamers. Such diversity provides a platform for a wide range of biomedical applications. The main synthesis tools for developing these conjugates are electrostatic adsorption, chemisorption, covalent binding and specific affinity interaction. One of the most promising techniques is covalent binding through the use of bifunctional linkers, whereby an anchor group is used to attach to the substrate whilst a functional group remains free to covalently bind to the target biomolecule. This technique mitigates the loss of biorecognition and/or bioactivity.

DNA hybridization has been successfully detected by LSPR [35]. Endo *et al.* [6] also monitored peptide nucleic acid (PNA)-DNA hybridisation using a gold-capped nanostructure layer substrate. The optical properties of the substrate were characterised through transmission measurements, achieving target DNA detection of concentrations as low as 1 fM.

Insulin is one of the most important indicators for diabetes diagnosis, a disease where 80% of related deaths occur in developing countries [40]. A novel polydimethylsiloxane (PDMS) microfluidic LSPR chip has been proposed to interrogate antibody-antigen reactions in real time with the chip later used to detect insulin levels in the real-time monitoring of insulin and anti-insulin antibody immunoreactions with a 100 ng/mL detection limit [41].

At present, 62% of people with dementia live in developing countries, and this is expected to rise to 71% by 2050 [42]. The development of an accurate diagnostic test for Alzheimer’s disease could help millions to obtain personalised treatment for their symptoms. A biosensor based on LSPR has been proposed by Haes *et al.* [43,44] to monitor the interaction between amyloid β-derived diffusible ligands (ADDL) and the anti-ADDL antibody, which are possibly implicated in Alzheimer’s disease. ADDL-functionalized Ag nanostructures were shown to display high selectivity to elevated concentrations of anti-ADDLs. The LSPR biosensor has the potential to become an accurate and economical alternative to traditional clinical assays.

Ultra-sensitive influenza detection through antigen-antibody interaction on a gold surface was successfully shown using LSPR [45]. In this work, an active immobilisation method was developed to facilitate the biosensing of avian influenza virus. The gold binding polypeptide (GBP)-fusion protein was bound onto the gold substrates by means of specific interaction. The GBP-fusion method allows the immobilisation of proteins in bioactive forms onto the gold surface without surface modification. This methodology could be extended to provide the detection of clinical diseases and other protein-protein interactions.

An LSPR bioanalysis method for the multi-array screening of antigen-antibody interactions was showcased by Endo *et al.* [7]. The method provided convenient, low-cost, label-free, specific and highly-sensitive detection, demonstrated using immunoglobulins (IgA, IgD, IgG, IgM), C-reactive protein and fibrinogen.

A membrane-based nanosensor has been reported to provide highly sensitive detection of peptide toxin using a core-shell structure nanostructure substrate [46]. The shell thickness was shown to play an important role in determining the extinction wavelength maximum. This feature is used for detecting the binding of peptide toxin melittin to a hybrid bilayer membrane (HBM) and electrochemically assessing its membrane-disturbing properties as a function of concentrations. This approach can be deployed to detect functionally-similar protein toxins and other membrane targeting peptides. One example is the *Staphylococcus aureus* enterotoxin B (SEB) protein toxin, a dangerous protein toxin that can cause nausea, vomiting, diarrhoea and even anaphylactic shock, where detection of SEB has been demonstrated for ng/mL levels [47].

*Salmonella* presents both a threat to public health and the risk of significant economic losses. In developing countries, nosocomial outbreaks are more prevalent, but the combination of increased agricultural activity and poor water quality is likely to increase the risks of foodborne *Salmonella*. The detection of bacterial pathogens remains challenging [48], with difficulties in repeatedly detecting the levels to an acceptable accuracy, and this is no different for LSPR-based detection [49]. More recently, the simultaneous detection of tuberculosis and *Schistosoma japonicum* (a parasite that affects around 210 million people worldwide, especially in Asian, African and Latin-American countries) using a simply fabricated substrate has been demonstrated in low serum concentrations (1:10,000) without the need for sample processing [50]. This marks a significant step in the detection of pathogens.

Lee *et al.* [51] propose a highly sensitive LSPR immunosensor for the detection of the HIV-1 virus. The surface of the Au nanostructure was modified with HIV-1 antibody fragments to measure various concentrations of HIV-1 particles quantitatively with a 200 fg/mL detection limit. Since this LSPR immunosensor has the advantages of rapid preparation, high sensitivity and selectivity, it is a promising approach for the screening of other viral particles.

The evaluation of cholesterol concentrations of the lipid membrane could be useful for the early detection of heart diseases and cancer. High levels of cholesterol in the lipid membrane can be associated with the initial formation of tumours, with two examples being breast and prostate cancer. A device based on LSPR has been proposed and simulated with FullWAVE^TM^ to measure cholesterol in the lipid membrane [52].

LSPR-based biosensors could also be used for the diagnosis of pregnancy-related conditions, such as preeclampsia, a hypertensive disorder occurring during pregnancy. Uric acid in urine caused by proteinuria can be used as a biomarker for preeclampsia, with concentrations above 0.4 mM being indicative of severe preeclampsia [53]. With 99% of the 500,000 maternal deaths each year occurring in developing countries and preeclampsia being one of the leading causes of maternal death worldwide, the sensitivity offered by an LSPR platform could allow for the early detection of such conditions, where early medical treatment could alter the course of progression.

Medical-orientated LSPR biosensors are expected to evolve mainly in the following directions: (i) higher sensitivity; (ii) detection of more complex systems beyond the molecular level, such as cellular-, intracellular- and tissue-level detection; (iii) a combination of current nanostructures with other materials; (iv) a combination of LSPR with other available transduction methods; and (v) overcoming the challenges of using LSPR sensing at the point of care and in field applications.

### 2.2. Agriculture and Environmental Monitoring

Since the adoption of the UN Millennium Declaration in 2000, tackling hunger across the world remains an important goal. Currently, around one billion people suffer from severe hunger, and this malnutrition makes people more susceptible to illness, poor growth and reduced work capacity, all leading to a depressed economy. Thus, food and agriculture can also be considered intrinsically linked with poverty.

With increasing population, changes in the climate and diminishing natural resources, agricultural productivity has again been put under severe strain. With the development of affordable biosensing technologies, agricultural output could be both increased and made more sustainable by improving the use of soil, water and fertiliser, as well as crop resistance to disease. Sustainability will come from better education, as well as the ability to monitor the environment for contaminants and pollutants in order to improve agricultural techniques.

The ability to monitor plant nutrients allows the yield and quality of crops to be improved. The current methods of analysing plant nutrients include: inductively-coupled plasma atomic emission spectroscopy (ICP/AES), X-ray fluorescence spectrometry (XRF), inductively-coupled plasma mass spectrometry (ICP-MS) and atomic absorption spectrometry (AAS), but all require the device to be situated in laboratory environments, due to several constraints, such as size, cooling and necessary gas supplies.

LSPR has been shown to offer enhanced sensitivity and limits of detection (LOD) for the laser-induced breakdown spectroscopy (LIBS) detection of calcium, iron, copper, sodium, potassium, cobalt, manganese and molybdenum [54]. This quick and simple technique highlights the power of an LSPR-based sensor for this application.

Agriculture is the single largest user of surface water and is both a cause and a victim of water pollution (quality) and contamination (health risk). Therefore the production/processing/distribution chain requires careful screening to allow pollutant/contaminant detection and the determination of the likely source.

Endocrine disrupting chemicals (EDCs), such as dioxins, industrial chemicals (e.g., PCBs (polychlorinated biphenyls)), agrochemicals (e.g., atrazine) and pharmaceuticals all accumulate *in vivo*, causing abnormalities in growth, reproduction, development, behaviour, immune response and the development of malignant tumours. Rapid screening for EDCs is highly needed. The detection of dioxins, PCBs and atrazine have been demonstrated using competitive and sandwich immunoassays [55]; PCBs have also been measured to the ppb level (50 pM) through LSPR combined with SERS [56].

Organophosphate (OP) pesticides pose a hazard to human health (eye pain, abdominal pain, paralysis and respiratory failure) and, as such, are an important target for detection. LSPR detection of OP agents at 0.234 ppb LOD has been demonstrated [57] by covalently coupling acetylcholinesterase (AChE) to nanostructures. OPs irreversibly bind with AChE, an essential enzyme in nerve impulse responses that causes OP’s toxicity.

The detection of chlorpyrifos and malathion, pesticides predominantly found in surface waters of developing countries, has been demonstrated to the ppb level using a simple UV-Vis spectrometer. The use of Na_2_SO_4_ improved the aggregation of the gold nanostructures, allowing amplified LSPR spectral shifts to be observed at low concentrations [58].

Mercury detection has been achieved by monitoring the affinity difference of Hg and Au nanostructures with DNA; increased concentrations of Hg cause changes in the aggregation state of Au nanostructures, and this yields stronger LSPR intensities [59]. This work is of significant importance given that concentrations of the majority of metals, regardless of being essential or non-essential, are toxic for living cells.

## 3. Challenges

Whilst LSPR shows plenty of promise for developing affordable, portable and highly-sensitive devices, there remain several challenges yet to overcome before such devices can come to fruition. Here, we outline some of the current issues as potential avenues to explore.

### 3.1. Nanostructures

Continual research is being conducted to develop biocompatible materials that possess a negative real and small positive imaginary dielectric constant [60], but at present, apart from gold nanostructures, few biocompatible materials are well established for LSPR measurements. Biomolecules are usually anchored to the gold nanostructures through thiol groups. However, it is reported that thiol-based self-assembled monolayers (SAMs) suffer defects due to thermal desorption and photooxidation that could bring unwanted changes in the LSPR signal [61,62]. Although alternative surface chemistries have been proposed for gold, it would be beneficial to develop other biocompatible materials capable of producing stable signals upon analyte binding. 

Another important challenge is to refine the fabrication processes in order to reliably reproduce structural nano-level parameters, such as size and shape; factors that greatly affect the reproducibility and reliability of systems to date. Whilst techniques like nanosphere lithography, electron-beam lithography and chemical synthesis, have assisted in the fabrication of more homogeneous nanostructures to some extent, progress is still required to retain affordability [10]. Several approaches have been proposed for the production of nanostructures anchored to low-cost polymers [63,64] and plastics [65], which could enable the production of large-area plasmonic structures for high-throughput screening. 

### 3.2. Application

A critical step for medical applications would be to develop generic strategies for testing biomarkers for as many diseases as possible; this would accelerate evolution to a stage where it could be used as a highly efficient diagnostic tool in developing countries. At present, extensive research has been done on the detection of a few diseases or disorders, but the application of LSPR for a wide range of diseases is still premature. Some fundamental issues that need further optimisation (and which are common to any label-free surface-based biosensors) include molecular fouling on the nanostructures and the inability of LSPR to distinguish analytes of similar functionality.

One area that could prove substantial is to optimise the use of LSPR systems within solutions. Organic molecules usually have a higher refractive index than the buffer mediums. This would also propel the use of microfluidic platforms, whereby multiplexing with other biosensing technologies would make it more attractive. In addition, the use of nanostructures inside single cells to measure spatial and temporal changes using LSPR would offer a fantastic platform to study intracellular and intercellular biochemistry [66,67].

Furthermore, given that thermal hotspots are always present in plasmonic structures, it would be interesting to develop LSPR sensors for *in vivo* biosensing with no need for sample manipulation. Tracking particles *in vivo* with LSPR would allow long-term mapping of biological systems, a key advantage over the use of labels, such as fluorophores. 

For both agriculture and environmental monitoring, sample preparation remains an area that requires improvement. In particular for water monitoring, the coexisting water/waste water constitutes makes reproducible results difficult [68].

### 3.3. Technology Transfer

LSPR-based biosensors have shown immense potential as a highly sensitive and selective platform for a variety of biosensing applications. In academia, LSPR-based biomolecular assays to study binding kinetics and conformational changes have been firmly established for some time now. Given the high sensitivity of plasmonic nanostructures to local changes in its refractive index, a large number of label-free LSPR biosensors utilising single nanostructures has also been demonstrated.

However, at present, there still remains a poor interface between academia and industry. These proofs of concept need to be converted into practical tools viable for commercialisation. Although a small number of high-performance systems are now commercially available, these are high cost (typically on the range of US $50,000), mostly suited for laboratory research use. In-house built systems using discrete components tend to be much cheaper ($5,000–$10,000), but are usually developed for specific academic research needs. There are several bottlenecks that hold up the technology transfer from academia to industry. Commercially viable point-of-care systems have increasing levels of prerequisites, such as portability, throughput and cost of instrumentation, which LSPR biosensors are still constrained to address [69].

As previously mentioned, a significant advancement of LSPR technology could be achieved by exploring its combination with other transduction techniques to offer simultaneous sensing methods on the same substrate; for instance, where gold nanostructures have been used as labels to identify analyte of interest or to amplify the signals [70,71] or through the use of complementary techniques, like chemiluminescence and electrochemical impedance spectroscopy. Such an integration could be used to validate and increase the confidence of measurements.

Spectrometric equipment is the area in which miniaturisation of the LSPR platform could be most noticeably achieved; previously, the major drawback of miniaturised spectrometers had been that they suffered from poor resolution [72]. 

A spectrometer typically consists of a light source, dispersive element, optical path and the detector. The dispersive element usually takes the form of a grating; here, the adoption of arrayed-waveguide gratings will allow for broader spectral ranges, whilst direct-writing of the photoresist during fabrication will allow for improved resolution [73] through more complicated and precise grating designs. Further work to improve the coupling of the optical path with the dispersive element should also improve the signal-to-noise ratio [74]. 

Arrays of either CMOS (complementary metal-oxide-semiconductor) photo diodes or CCDs (charge-coupled devices) offer the most cost-effective platforms for the detection element. The fact that CCD technology has been pushed, predominantly by the digital camera market, could mean they remain the primary choice. Should a better dynamic range or an improved noise floor be needed, then detector cooling through the use of thermoelectric coolers [75] can be implemented. Finally, recently developed silicon photomultipliers [76] offer a promising horizon in terms of performance [77], provided that the affordability is improved.

As mentioned, the success of the digital camera market has driven the cost of quality photodetectors down to such a point that they are commonly found on mobile telephones or smartphones. Importantly, an LSPR platform comprised of a camera phone, LED source, cuvette and lens adaptor was demonstrated by Roche *et al.* [78]. This marks a significant step towards a portable, affordable LSPR platform by using the integrated photodetection capability of a smartphone. Furthermore, another optical biosensor utilising a camera phone was recently demonstrated by Dutta *et al.* [79], where a smartphone was used to perform evanescent wave-coupled spectroscopy.

An alternative to this approach would be to use a smartphone for data acquisition, processing, storage, display and transmission, leaving a custom device for the sample processing, microfluidics and spectroscopy. Such a device could be better tailored for high sensitivity and throughput. 

Since smartphones are now ubiquitous in the developed world (and a rapidly expanding market in developing countries), they offer an affordable alternative to dedicated hardware for the aforementioned tasks. Through the use of custom software that can be continually updated, a range of peripheral biosensors can be operated by untrained personnel allowing a quick read-out and also the potential to transmit data to central hubs through the cellular network (a strategy that looks promising for developing countries [80]). However, at present, there is no standardisation of the data format or connection protocol for mobile biosensors, and this is an important factor that needs to be addressed [81]. 

Finally, wider adoption of established manufacturing processes such as very-large-scale integration (VLSI), CMOS and microelectromechanical systems (MEMS) would aid the integration of sample preparation and microfluidics. This would make array-based, high-throughput LSPR sensors a realistic proposition for the expansion into mass production of low-cost portable devices. Medium resolution systems costing $100–$500 could effectively be developed with currently available discrete components, but both price and resolution are expected to significantly improve in the short term upon suitable technological integration. Depending on the complexity of the individual chips, the production cost per analysis could easily come down to less than $1.

## 4. Summary

Clearly, LSPR presents itself as an encouraging platform for lab-on-a-chip point-of-care devices. A wide range of applications have already been demonstrated, albeit in a state of infancy. Although further work is needed on the sensor substrate fabrication and functionalisation to improve reproducibility and selectivity, high sensitivity has been widely achieved.

The most significant challenge may be considered that of translating these research efforts into real product availability through the active collaboration of universities, research centres, industries and private equity investors. The intrinsic scale of LSPR-based sensors means that only small sample volumes are required, lending itself to miniaturised devices incorporating microfluidics, sample preparation, sample processing and any necessary electronics.

Miniaturisation will come from both the advent of sensor substrate fabrication processes that are viable for mass production and the integration of VLSI, CMOS and MEMS techniques for integrated sample preparation and microfluidics. 

The use of smartphones instead of expensive dedicated hardware for the tasks of data acquisition, processing, storage and read-out is a promising proposition given that they can also be used to transmit data back to central processing hubs. This would allow wide areas to be effectively managed, preventing the rapid spread of disease and the ability to better understand isolated environmental issues.

The further miniaturisation and mass production of LSPR systems would improve the viability of use in remote areas, where robust and high-throughput biosensors for medical diagnosis and environment monitoring are highly needed. LSPR sensors may well be the ideal candidates for the future low-cost detection of endemic diseases and the monitoring of agricultural activity and environmental sustainability in developing countries. 
